# Simplified Near-Degenerate Four-Wave-Mixing Microscopy

**DOI:** 10.3390/molecules26175178

**Published:** 2021-08-26

**Authors:** Jianjun Wang, Xi Zhang, Junbo Deng, Xing Hu, Yun Hu, Jiao Mao, Ming Ma, Yuhao Gao, Yingchun Wei, Fan Li, Zhaohua Wang, Xiaoli Liu, Jinyou Xu, Liqing Ren

**Affiliations:** 1 School of Energy Engineering, Yulin University, Yulin 719000, China; wjj@yulinu.edu.cn (J.W.); zhangxi@yulinu.edu.cn (X.Z.); 1805210110@yulinu.edu.cn (J.D.); huxing@yulinu.edu.cn (X.H.); huyun@yulinu.edu.cn (Y.H.); 1905210110@yulinu.edu.cn (J.M.); 1805210145@yulinu.edu.cn (M.M.); 1905310102@yulinu.edu.cn (Y.G.); weiyingchun@yulinu.edu.cn (Y.W.); hypedynamics@yulinu.edu.cn (F.L.); wangzhaohua123@yulinu.edu.cn (Z.W.); liu_xiaoli@yulinu.edu.cn (X.L.); 2 South China Academy of Advanced Optoelectronics, South China Normal University, Guangzhou 510006, China

**Keywords:** near-degenerate, four-wave-mixing, microscopy

## Abstract

Four-wave-mixing microscopy is widely researched in both biology and medicine. In this paper, we present a simplified near-degenerate four-wave-mixing microscopy (SNDFWM). An ultra-steep long-pass filter is utilized to produce an ultra-steep edge on the spectrum of a femtosecond pulse, and a super-sensitive four-wave-mixing (FWM) signal can be generated via an ultra-steep short-pass filter. Compared with the current state-of-the-art FWM microscopy, this SNDFWM microscopy has the advantages of simpler experimental apparatus, lower cost, and easier operation. We demonstrate that this SNDFWM microscopy has high sensitivity and high spatial resolution in both nanowires and biological tissues. We also show that the SNDFWM microscopy can achieve an ultra-sensitive detection based on the electron-resonance effect. This method might find an important application in tracking of nano drugs in vivo.

## 1. Introduction

The continuous progress of optical microscopy is of great significance to the development of biomedicine. High sensitive fluorescence microscopy has been widely used in basic research at the nanoscale [1,2,3].

FWM has been widely used in nonlinear optical microscopy for decades, such as coherent anti-Stokes Raman scattering (CARS) [4,5,6] and stimulated Raman scattering (SRS) [7,8,9]. These two kinds of label-free and non-invasive microscopy have been studied widely. CARS microscopy, developed in 1982, is a nonlinear optical FWM process, whose signal strength is nearly 100,000 times higher than that of spontaneous Raman signals [10]. The CARS microscope was optimized by Xie’s group at Harvard University. This microscope is composed of a laser-scanning microscope, which realizes the scanning of externally introduced laser and the collection of CARS signals through a simple modification of commercial confocal microscopy [11]. Two pulsed lasers, called pump and Stokes, which coincide in space and synchronize in time, are used as the excitation light source. The time overlap of two laser pulse sequences of different wavelengths can be achieved in a variety of ways. One approach is to use two titanium: sapphire lasers to generate a pump beam and a Stokes beam of the right wavelength, respectively. An external synchronizer synchronizes the two pulses, and an optical delay line allows the two pulses to overlap precisely. The advantage of this method is that the wavelengths of the two laser beams can be tuned in a large spectral range. Its disadvantage is that the use of the external synchronizer not only increases the cost, but also makes it difficult to maintain the stability of synchronization, which increases the difficulty of experiment, thus restricting the application of this method. Another method uses an optical parametric oscillator (OPO) as the key device for wavelength tuning; this has gradually become the mainstream configuration method of the CARS system over the last two decades [12]. The main reason is that this method eliminates the need for synchronous control of two independent pulse lasers and simplifies the experimental setup. However, Silberberg’s research group implemented a single-beam CARS microscope by using the pulse shaping method [13]. Later, the team further simplified the single-beam CARS microscope system by replacing the pulse shaper with a resonant photonic crystal plate or notch filter [14,15]. Recently, researchers found that the resonant photonic crystal plate or notch filter can be removed from the system by using the ultra-steep filter, proving the simplest CARS microscope to date [16].

Single-beam near-degenerate four-wave-mixing microscopy can provide two imaging contrast mechanisms, with nearly perfect phase-matching conditions, providing an effective imaging contrast mechanism [17]. In this paper, we present a simpler near-degenerate FWM microscopy by replacing the complex pulse shaper used in a previous study with a compact and low-cost long-pass filter, an ultra-steep long-pass filter (ULPF). This ULPF produces a sharp “edge” on the spectrum of the incoming femtosecond laser, achieving an infinitely close FWM signal on the spectrum to the incident laser, and thus generating a super-strong FWM signal. Simplifying the experimental apparatus and its operation, the cost of such a system is also lowered. We call this method a simplified near-degenerate four-wave-mixing (SNDFWM) microscope system.

## 2. Theory

In the process of four-wave-mixing, when the frequency of the detection wave and the pump light are the same, that is, when the frequency of the four waves are the same, it is called degenerate four-wave mixing. In practical applications, detection light and pump light often have a certain frequency difference, and when their frequency difference is much smaller than any of the frequencies involved in mixing, it is the so-called near-degenerate four-wave-mixing process. The third-order nonlinear polarizability tensor in the process of near-degenerate four-wave-mixing is described as [18]:

(1) $$\chi^{\left(3\right)}(-\omega_{4})=\frac{N}{\hbar^{3}}P_{F}\sum_{m,l,n}\frac{\mu_{km}\mu_{ml}\mu_{\ln}\mu_{nk}}{\left(\omega_{mk}-\omega_{1}-i\gamma_{mk})(\omega_{lk}-\omega_{1}-\omega_{2}-i\gamma_{lk})(\omega_{nk}-\omega_{4}-i\gamma_{nk}\right)}$$

where P_F_ is the full permutation operator, *µ* is the transition dipole moment, *ω* is the energy difference between the corresponding energy levels, and *γ* is the uniform linewidth associated with the corresponding electron transition or vibrational transition. In Equation (1), the third-order nonlinear polarizability tensor will reach the maximum value when the real part of the value inside the brackets on the denominator is the minimum (or equal to zero), especially when the incident light reaches resonance with the sample. For example, such resonance enhancement occurs when the wavelength of the incident light coincides with the absorption peak of the fluorescent material.

The SNDFWM spectrum measured in the experiment can be quantitatively described by continuous integral [17]:

(2) $$I(\omega_{4})\propto {\left|P^{\left(3\right)}(\omega_{4})\right|}^{2}\propto {\left|\int_{-\infty }^{\infty }d\omega_{1}\int_{-\infty }^{\infty }d\omega_{2}\int_{-\infty }^{\infty }d\omega_{3}\chi^{\left(3\right)}(-\omega_{4})E(\omega_{1})E^{*}(\omega_{2})E(\omega_{3})\right|}^{2}$$

where $\omega_{4}=\omega_{1}-\omega_{2}+\omega_{3}$.

## 3. Experimental Equipment

The main function of the pulse shaper is to filter out some wavelength components of the incident laser so that the spectrum of this laser can reach the near-degenerate condition. Besides being complex to adjust, the pulse shaper is also relatively expensive. With the progress of science and technology, a kind of ultra-steep long-pass filter has come into being. This filter not only has the function of a long-pass filter, but can also cut off the unneeded wavelength components very steeply. Therefore, this filter can replace the pulse shaper used in the near-degenerate four-wave-mixing scheme. This not only simplifies the experimental equipment, but also realizes the low-cost and easy-to-operate microscopic technique.

**Figure 1:**
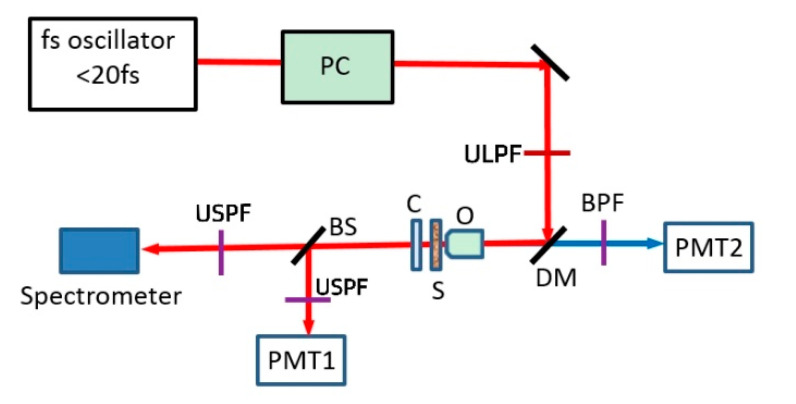
Schematic diagram of the SNDFWM microscope. PC: pulse compressor; fs: femtosecond; ULPF: ultra-steep long-pass filter; O: objective lens; S: Sample; C: condenser lens; DM: dichromatic mirror; BPF: band-pass filter; PMT: Photomultiplier tube; BS: beam splitter.

The SNDFWM microscope device is shown in Figure 1. An ultra-short pulse with a central wavelength of 800 nm, a repetition frequency of 80 MHz, and a pulse width of 20 fs was used to compensate the laser dispersion at the sample position through the pulse compressor, and then truncated steeply by the ultra-steep long-pass filter (ULPF). After that, a dichromatic mirror was reflected and introduced into the microscopic objective lens to focus the sample placed on the 2-D precisely-controlled stage. The reflected part of the scattered signal is collected by the same objective lens and then filtered by a band-pass filter. Finally, it is introduced into a photomultiplier tube (PMT2) for two-photon excited fluorescence (TPEF) imaging. The transmitted part of the scattered signal is collected by a condenser lens and divided into two channels by a beam splitter mirror. One way enters the high-resolution spectrometer through an USPF for SNDFWM spectrum measurement, and the other way enters into another photomultiplier tube (PMT1) after a second USPF for SNDFWM imaging. The sample is placed on a two-dimensional movable high-precision platform.

## 4. Results and Discussion

**Figure 2:**
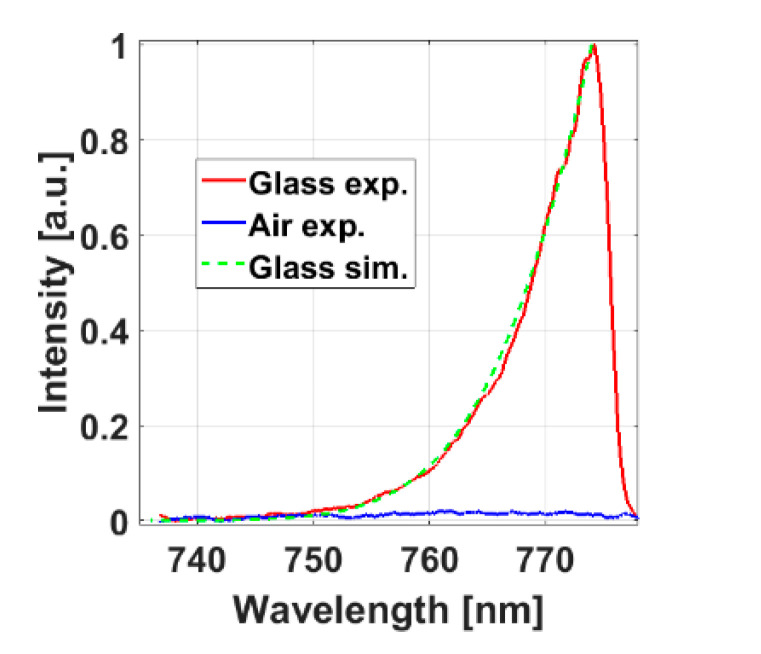
System test via the SNDFWM microscope. The solid red line indicates the experimental measurement of SNDFWM spectrum of glass. The blue solid line indicates the experimental measurement of SNDFWM spectrum of air. The green dotted line indicates the theoretical simulation of SNDFWM of glass.

First, glass and air were used as samples for experimental detection of the SNDFWM microscope as shown in solid red and blue lines in Figure 2, respectively. Equation (2) was used for theoretical simulation calculation of the SNDFWM spectrum of the glass, as shown by the dashed green line in Figure 2. The solid blue line in Figure 2 shows that the air does not generate a FWM signal at all. The results in Figure 2 show that the theoretical simulation results are in good agreement with the experimental results. Therefore, both the theoretical simulation and the experimental measurement of the corresponding samples prove the feasibility of the system.

**Figure 3:**
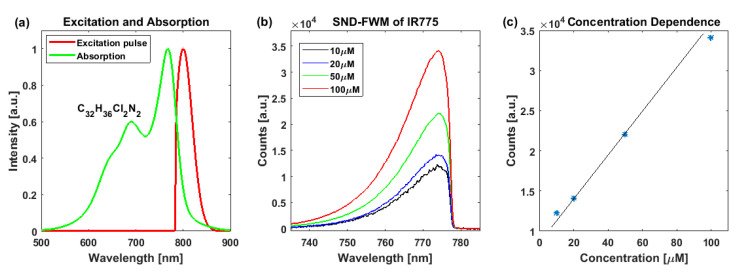
Resonance-enhanced SNDFWM spectrum of IR775 solution. (**a**) Spectrum of the excitation pulse (red) and absorption spectrum (green) of IR775 solution; (**b**) SNDFWM spectra of IR775 solution with different concentrations; (**c**) Concentration dependence of SNDFWM signals.

Secondly, our experimental results show that the electron-resonance effect can be used to achieve ultra-sensitive spectral detection. This is especially important for samples that have only spectral absorption but no fluorescent signal, as such samples are difficult to see with a fluorescence microscope. According to Equation (1), when the wavelength of the incident laser is within the absorption spectrum of the sample, one can greatly enhance the FWM signal by using the effect of electron-resonance. Figure 3 shows the ultra-sensitive detection of a solution of bleach IR775 that resonates at 775 nm. The results in the figure show that when the incident light power is 1 mW and the integration time is 100 ms, the detection sensitivity was similar to the previous results [17]. Therefore, the proposed SNDFWM microscope can also achieve ultra-high sensitive detection.

**Figure 4:**
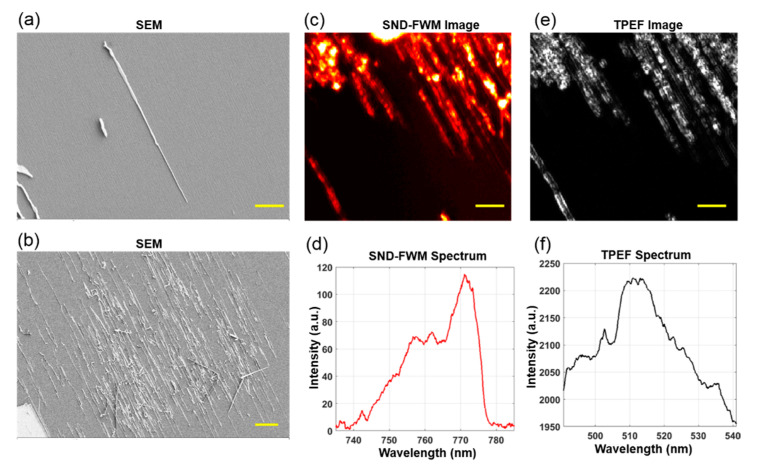
SNDFWM microscopic imaging of CdS nanowires. (**a**) Scanning electron microscopy (SEM) images of CdS nanowires with a scale of 2 µm; (**b**) SEM images of nanowires with a scale of 10 µm; (**c**) SNDFWM image of CdS nanowires with a scale of 5 µm; (**d**) SNDFWM spectrum of CdS nanowires; (**e**) Two-photon excited fluorescence (TPEF) image of CdS nanowires with 5 µm; (**f**) TPEF spectrum of CdS nanowires. The pixel size of the SNDFWM and TPEF images is 0.5 µm.

To further demonstrate the high spatial resolution of the proposed system, we employed an objective lens (Olympus ×60/1.2) to perform imaging of the CdS nanowires (with a transverse mean diameter of about 93 nm), as shown in Figure 4. CdS nanowires were prepared by using the chemical vapor deposition [19] method. The growth of CdS nanowires was performed in a home-build two-zone horizontal tube furnace. In a typical synthesis, about 0.12 g CdS power (99.99%, Sigma-Aldrich, St. Louis, MO, USA) evaporated at 860 °C under 300–400 mbar served as the precursor. The CdS vapor was then carried to the M-plane sapphires (8 × 8 mm^2^) pre-coated with Au catalysts (average 50 nm) by a high-purity N_2_ gas flow at a rate of 400 sccm. The temperature around the sapphire was maintained at 560–600 °C for 30 min to enable the desired nanowire growth. Figure 4a,b are images of CdS nanowires at different magnifications under scanning electron microscopy, respectively. Figure 4c,d are SNDFWM imaging and the corresponding spectral images, respectively. It is shown that this method can be utilized to perform imaging of a single nanowire. The single-nanowire sensitivity of SNDFWM microscopy means that such an approach is a reliable technique for examining the electronic structure of materials at the nanoscale [20]. Therefore, as long as the wavelength of the incident light is short enough (e.g., ultraviolet laser) and the numerical aperture of the microscopic objective lens is large enough, a microscopic image with higher spatial resolution can be achieved. 

**Figure 5:**
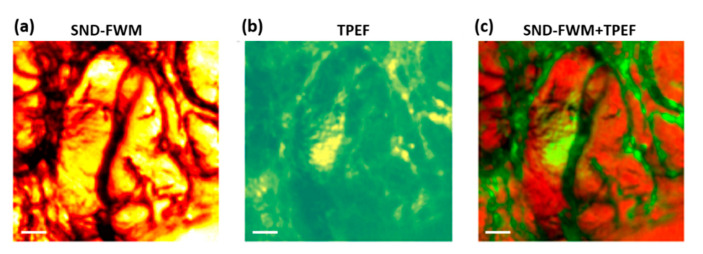
Microscopic imaging of mouse brain using SNDFWM microscope. (**a**) SNDFWM image; (**b**) TPEF image; (**c**) Overlaid image of SNDFWM and TPEF. The white scale is 20 µm, the pixel-dwell time is 200 microseconds, the pixel size is 1 µm, and the incident laser power is 1 mW.

Finally, we apply the proposed system to high spatial resolution microscopic imaging of mouse brain tissue samples, as shown in Figure 5. The preparation methods of mouse brain tissue samples were consistent with those reported in the literature [6]. Fresh mouse brain tissues were commercially procured (Zyagen) and pre-mounted on charged glass slides. The samples were shipped on dry ice and stored at −80 °C. They were thawed for 10 min, and then washed twice in PBS to remove debris and residual cutting media before imaging. The tissues were kept wet with PBS and sandwiched with a glass coverslip over the sample and the glass slide, and then sealed with nail polish. Figure 5a is the SNDFWM image of mouse brain tissue, while Figure 5b is a two-photon excited fluorescence (TPEF) image obtained at the same time. Figure 5c is the result of the superposition of a FWM and reflected TPEF image. The spatial distribution of blood vessels and white matter in normal brain tissue can be seen clearly in this image. This is because the TPEF is more sensitive to blood, while the FWM is more sensitive to the white matter. During the experiment, we only used the numerical aperture of 0.4 objective lens (Newport ×20/0.4), and we only used 1 mW incident light power and 200 ms pixel-dwell time, which shows that the SNDFWM microscope has high spatial resolution and high sensitivity. This is of great scientific significance for biomedical applications.

Compared with the previous study [17], we replaced the pulse-shaping system with a compact ULPF, simplifying the NDFWM microscope system significantly. This simplified setup is smaller, cheaper, and easier-to-operate. In comparison with our previous work [15], less excitation energy is needed to excite the FWM signals and the imaging speed is much faster. This method might find significant application in tracking nanodrugs in vivo [21].

## 5. Conclusions

In conclusion, we have presented a well-designed SNDFWM microscope device. We successfully observed the high-resolution multimodal imaging of brain tissue of mice, and FWM imaging of a single nanowire. The microscope system device is not only simple and low-cost, but also easy-to-operate. These advantages are the guarantee that SNDFWM microscopy can be widely used in the field of biomedical imaging and nanomaterials.

## Data Availability

The data presented in this study are available on request from the corresponding author.
